# Editorial for Special Issue: Neuroproteomics

**DOI:** 10.3390/proteomes7020024

**Published:** 2019-05-31

**Authors:** Kenneth R. Williams, Angus C. Nairn

**Affiliations:** 1 Yale/NIDA Neuroproteomics Center, New Haven, CT 06511, USA; 2 Molecular Biophysics and Biochemistry, Yale University School of Medicine, New Haven, CT 06511, USA; 3 Department of Psychiatry, Yale School of Medicine, Connecticut Mental Health Center, New Haven, CT 06511, USA

Recent advances in mass spectrometry (MS) instrumentation [1,2], especially in MS resolution and scan rate enable the quantitation of expression of more than 15,000 proteins (>12,000 genes) from mammalian tissue samples [3,4]. These advances have opened the door to the proteome and already are having an impact that extends from biology to clinical proteomics. With no theoretical limits in sight—with regard to further improvements in MS instrumentation and improved peptide identification algorithms and bioinformatics—the future of MS-based, quantitative proteomics is incredibly promising and exciting. Indeed, new chemical labeling technologies that incorporate multiple isobaric tags now enable concurrent analyses of up to 11 different samples using commercially available reagents [5]. While these methods are beginning to be applied to neuroproteomics, the central nervous system (CNS) poses unique challenges to quantitative proteomics that begin with the immense level of cellular and sub-cellular heterogeneity. The human CNS has ~100 billion neurons, each with 10,000 to 100,000 synaptic connections; and even larger numbers of glial cells. Moreover, there is a large variety in cell morphology with individual neurons typically being intermingled in close contact with several different types of neurons and with axonal projections from an individual neuron often projecting over relatively long distances. Given that it is now clear that each of the ~500–1000 individual types of nerve cells exhibit distinct patterns of gene expression [6,7], it is critically important to develop and publish the technologies and methodologies needed to enable quantitative MS/proteomic analyses of specific neuronal cell types and their organelles. This topic is reviewed by Wilson and Nairn [8], and Wang and Savas [9], who highlight that cell-type-specific analysis has become a major focus for many neuroscience investigators. While the whole brain or large regions of brain tissue can be used for proteomic analysis, the useful data that can be gathered is limited because of cellular and sub-cellular heterogeneity. Analysis of mixed populations of distinct cell types not only limits our understanding of where a particular protein expression change might have occurred, it also minimizes our ability to detect significant changes in protein expression and/or modification levels due to issues related to dilution effects and low signal to high noise. Moreover, isolation of specific cell types can be challenging due to their nonuniformity and complex projections to different brain regions. In addition, many analytical techniques used for protein detection and quantitation remain insensitive to the low amounts of protein extracted from specific cell populations. Despite these challenges, methods to improve the proteomic yield and increase resolution continue to develop at a rapid rate. 

The review by Wang and Savas [9], and the article by Roy et al. [10], show that proteomic heterogeneity in the brain extends beyond the cell type to synaptic and postsynaptic density (PSD) proteomes, respectively. Different types of synapses in the brain have highly specialized neuronal cell-cell junctions, with both common and distinct functional features that arise from their individual synaptic protein compositions. Even a single neuron can have several different types of synapses that each contain hundreds or even thousands of different proteins. While MS/proteomic analyses provide a powerful approach for characterizing different types of synapses and to potentially identify disease-causing alterations in synaptic proteomes, the value of most synaptic proteomic analyses that have been published are also limited by the molecular averaging of proteins from the multiple types of neurons and synapses that often have been analyzed together. In their review, Wang and Savas [9] summarize a wide range of currently available technologies for analyzing neuron-type specific and synapse-type specific proteomes and discuss strengths and limitations of each of these technologies for successfully addressing the “averaging problem”.

The study by Roy et al. [10] was designed to determine if the synaptic proteome differs across anatomically distinct brain regions. Postsynaptic protein extracts were isolated from seven forebrain and hindbrain regions in mice and their compositions were determined using MS/proteomics. Across these regions 74% of proteins showed differential expression with each region having a distinctive composition. These compositions correlated with the anatomical regions of the brain and their embryological origins. Proteins in biochemical pathways controlling plasticity and disease, protein interaction networks, and individual proteins involved with cognition all showed differential regional expression. In toto, the Roy et al. [10] study showed that interconnected regions have characteristic proteome signatures and that diversity in synaptic proteome composition is an important feature of mouse and human brain structure.

Both Wilson and Nairn [8], and Wang and Savas [9], described the use of in situ proximity labeling methods to identify protein-protein interactions within discrete cellular compartments. As an example of the use of this technology, the Cijsouw et al. [11] article describes the use of this approach to map the proteome of the synaptic cleft, which is the space between two neurons at a nerve synapse. Cijsouw et al. [11] used a peroxidase-mediated proximity labeling approach with the excitatory-specific synaptic cell adhesion protein SynCAM 1 fused to horseradish peroxidase (HRP) as a reporter in cultured cortical neurons. This reporter marked excitatory synapses, as detected by confocal microcopy, and was localized in the edge zone of the synaptic cleft, as determined using 3D dSTORM super-resolution imaging. Proximity labeling with a membrane-impermeant biotin-phenol compound limited labeling to the cell surface, and label-free quantitation (LFQ) MS combined with ratiometric HRP tagging of membrane vs. synaptic surface proteins was used to determine the protein composition of excitatory clefts. Novel cleft proteins were identified and one of these, Receptor-type tyrosine-protein phosphatase zeta, was independently validated using immunostaining. The Cijsouw et al. [11] study supports the use of peroxidase-mediated proximity labeling for quantifying changes in the synaptic cleft proteome that may occur in diseases such as psychiatric disorders and addiction.

The ability of targeted mass spectrometry technologies to quantify the same proteins in multiple samples with the highest possible sensitivity, quantification precision, and accuracy [12] makes these technologies ideal for analyzing the small amounts of protein that result from the use of fluorescence-activated cell sorting (FACS), laser capture microdissection (LCM), and other technologies described by Wilson and Nairn [8] and Wang and Savas [9] to analyze single cell types and region-specific synaptic proteomes. In regard to the latter, there is increasing interest especially in understanding the functions of proteins in the PSD because of their potential involvement in a wide variety of neuropsychiatric disorders including autism spectrum disorder (ASD) [13,14,15] and schizophrenia [16]. As described in the Wilson et al. [17] article, the PSD is an electron-dense region located just beneath the postsynaptic membrane of excitatory glutamatergic synapses, which is involved in a wide range of cellular and signaling processes in neurons. Biochemical fractionation combined with MS/proteomics analyses has enabled cataloging of the PSD proteome. However, since the PSD composition may change rapidly in response to stimuli, robust and reproducible technologies are needed to quantify changes in PSD protein abundance. Using a data-independent acquisition (DIA) approach on PSD fractions isolated from mouse cortical brain tissue and a pre-determined spectral library, Wilson et al. [17] quantified over 2,100 proteins. In addition, Wilson et al. [17] designed a targeted, parallel reaction monitoring (PRM) assay with heavy-labeled, synthetic internal peptide standards to rigorously quantify 50 PSD proteins. Wilson et al. [17] suggest that the PSD/PRM assay is particularly appropriate for validating differentially expressed proteins identified by the DIA assay.

Despite the challenges in carrying out quantitative MS/proteomics analyses on neural tissues, sufficient progress has been made that neuroproteomics is increasingly being used to improve diagnosis and staging, and to help develop better treatments for a broad range of neurological diseases. With the number of Americans with Alzheimer’s disease (AD) expected to increase from an estimated 5 million in 2014 to nearly 14 million in 2060 [18] and with the costs of treating this disease expected to increase from $190 billion in 2019 to between $379 and $500 billion annually in 2040 [19]; there is considerable interest in finding more sensitive and specific diagnostic tools for this devastating disease that is now the 5th leading cause of death among adults aged 65 years or older [20]. As described in the review article by Carlyle et al. [21], neurodegenerative dementias like AD are highly complex diseases. While most can be diagnosed by pathological analyses of the postmortem brain, clinical disease symptoms often involve overlapping cognitive, behavioral, and functional impairments that pose diagnostic challenges in living patients. As global demographics shift towards an aging population, especially in developed countries, clinicians need more sensitive and specific assays that can be carried out on readily available bodily fluids, such as sera or plasma to diagnose, monitor, and treat neurodegenerative diseases. The Carlyle et al. [21] review provides an overview of how contemporary MS/proteomic and state of the art capture-based technologies can contribute to the discovery of improved biofluid biomarkers for neurodegenerative diseases, and the limitations of these technologies. The Carlyle et al. [21] review also discusses technical considerations and data processing approaches for achieving accurate and reproducible findings and reporting requirements to help improve our ability to compare data from different laboratories. 

As reviewed in the Lutz and Peng [22] article, characteristic features of AD include protein aggregates such as amyloid beta plaques and tau neurofibrillary tangles in the patient’s brain. Determining the complete composition and structure of the protein aggregates in AD can increase our understanding of the underlying mechanisms of AD development and progression. The Lutz and Peng [22] review summarizes the use of LCM—which was also reviewed in the Wilson and Nairn [8], and Wang and Savas [9] articles—and the differential extraction approaches needed to achieve deep profiling of the aggregated proteomes in AD samples, and discusses the resulting novel insights from these analyses that may contribute to AD pathogenesis.

A number of articles in this Special Issue are focused on addictive diseases. To grasp the importance of this area of research one has only to glance at data in the Surgeon General’s Report [23] for 2015 that states that 66.7 million people in the U.S. reported binge drinking in the past month and 27.1 million people were current users of illicit drugs or misused prescription drugs. While the accumulated costs of addiction to the individual, family, and the community are staggering, with the economic burden of prescription opioid misuse alone in the U.S. amounting to $78.5 billion annually [24], the most devastating consequences are the tens of thousands of fatalities each year as a result of substance abuse. In this regard, alcohol misuse contributes to 88,000 deaths annually in the U.S. In addition, in 2014 there were 47,055 drug overdose deaths, including 28,647 people who died from an opioid overdose—more than in any previous year. As reviewed by Natividad et al. [25], drug addiction is a complex disease caused by abnormally regulated molecular signaling across several brain reward regions. Due to our incomplete understanding of the molecular pathways that underlie addiction, there currently are only a few treatment options. Recent research suggests that addiction results from the overall impact of many small changes in molecular signaling networks that include neuropeptides (neuropeptidome), protein-protein interactions (interactome), and protein post-translational modifications (PTMs) such as protein phosphorylation (phosphoproteome). Advances in MS/proteomics instrumentation and technologies are increasingly able to identify the molecular changes that occur in the reward regions of the addicted brain and to translate these findings into new treatments. In their review Natividad et al. [25] provide an overview of MS/proteomics approaches for addressing critical questions in addiction neuroscience and they highlight recent innovative studies that demonstrate how analyses of the neuroproteome can increase our understanding of the molecular mechanisms that underlie drug addiction.

As discussed by Pena et al. [26], the treatment of chronic pain has been challenging as the most effective treatment that uses opiates has many unwanted side effects. For example, treatment with morphine quickly leads to µ opioid receptor (MOR) desensitization and the development of morphine tolerance. MOR activation by the peptide agonist, [D-Ala2, N-MePhe4, Gly-ol]-enkephalin (DAMGO), leads to G protein receptor kinase activation, β-arrestin recruitment, and subsequent receptor endocytosis, which does not occur with morphine. However, MOR activation by morphine induces receptor desensitization in a protein kinase C (PKC)-dependent manner. While PKC inhibitors decrease receptor desensitization, reduce opiate tolerance, and increase analgesia; the mechanism of action of PKC in these processes is not well understood. The challenges in establishing a role for PKC result, in part, from the inability to identify PKC targets. To meet this challenge Pena et al. [26] generated a conformation state-specific anti-PKC antibody that preferentially recognizes the active state of this kinase. Using this antibody to isolate PKC substrates and MS/proteomics to identify the resulting proteins, Pena et al. [26] determined the effect of morphine treatment on PKC targets. They found that morphine strengthens the interactions of several proteins with active PKC. Pena et al. [26] describe the role of these proteins in PKC-mediated MOR desensitization and analgesia, and they propose a role for some of these proteins in mediating pain by tropomyosin receptor kinase A (TrKA) activation. Finally, Pena et al. [26] discuss how these PKC interacting proteins and pathways might be targeted for more effective pain treatment.

As described by Mervosh et al. [27], there is increasing interest in the role that neuroimmune interactions play in the development of psychiatric illness, including addiction. This raises the possibility that targeting neuroimmune signaling pathways may be a viable treatment for substance use disorders. Calipari et al. [28] recently determined that granulocyte-colony stimulating factor (G-CSF), which is a cytokine, is up-regulated following chronic cocaine use [11]. Peripheral injections of G-CSF potentiated the development of locomotor sensitization, conditioned place preference, and self-administration of cocaine, and blocking G-CSF function in the mesolimbic dopamine system abrogated the formation of conditioned place preference. Despite these effects on behavior and neurophysiology, the molecular mechanisms by which G-CSF brings about these changes in brain function are unclear. In the Mervosh et al. [27] study, mice were treated with repeated injections of G-CSF, cocaine, or both, and changes in protein expression in the ventral tegmental area (VTA) were examined using 10-plex tandem mass tag (TMT) labeling coupled with LC-MS/MS analyses. Repeated G-CSF treatment resulted in differential expression of 475 proteins in multiple synaptic plasticity and neuronal morphology signaling pathways. While there was significant overlap in the proteins that were differentially expressed in each of the three treatment groups, injections of cocaine and the combination of cocaine and G-CSF also resulted in subsets of differentially expressed proteins that were unique to each treatment group. This study identified proteins and pathways that were differentially regulated by G-CSF in an important limbic brain region and will help guide further study of G-CSF function and its evaluation as a possible therapeutic target for the treatment of drug addiction.

As summarized by Natividad et al. [25], MS/phosphoproteomics has provided addiction researchers with a useful tool for measuring changes in activated states that may be devoid of changes in the corresponding protein levels. The phosphorylation of serine, threonine and tyrosine residues is one of the most common post-translational modifications (PTMs) that can act as a molecular switch and modulate a wide range of biological activity including signal transduction, cell differentiation/proliferation, protein-protein and protein-gene interactions, and subcellular localization. Natividad et al. [25] note that many hypotheses invoke differential protein phosphorylation to control the activities of key regulators of gene transcription (e.g., the cAMP response element-binding protein, delta fosB), membrane receptors (e.g., GluA1) and other important binding partners (e.g., transmembrane α-amino-3-hydroxy-5-methyl-4-isoxazolepropionic acid (AMPA) receptor regulatory proteins as summarized by Park [29]) that modulate neuroplasticity. Indeed, there are several hundred eukaryotic kinases and phosphatases that have a broad range of substrate targets [30]. Since a substantial component of receptor-mediated neuronal signaling involves modulation of the activities of kinases and phosphatases, large-scale phosphoproteome profiling is a key technology that can provide unique information into the roles of protein phosphorylation in addiction.

As summarized by Park [29], strengthening and weakening of synaptic transmission (i.e., synaptic plasticity) provides a critical mechanism for many brain functions including learning, memory, and drug addiction. Long-term potentiation (LTP) and depression (LTD) are well-characterized models of synaptic plasticity that can be regulated by changes at presynaptic (e.g., changes in the release of neurotransmitters) and postsynaptic (e.g., changes in the number and properties of neurotransmitter receptors) sites. As shown in cellular models of synaptic plasticity, changes in the post-synaptic activity of the AMPA receptor (AMPAR) complex mediates these phenomena. In particular, Park [29] notes that protein phosphorylation plays a key role in controlling synaptic plasticity, for example, Ca^2+^/CaM-dependent protein kinase II (CaMKII) in hippocampal LTP. The Park [29] review summarizes studies on phosphorylation of the AMPAR pore-forming subunits and auxiliary proteins including transmembrane AMPA receptor regulatory proteins (TARPs) and discusses its role in synaptic plasticity.

Just as protein phosphorylation plays a key role in the molecular mechanisms underlying drug addiction, the articles by Bertholomey et al. [31] and Miller et al. [32] indicate that this PTM also plays an important role in alcohol use disorders (AUDS) and nicotine addiction, respectively. Bertholomey et al. [31] describe how early life stress is associated with an increased risk of developing AUDs. Although the neurobiological mechanisms underlying this effect are not well understood, abnormal glucocorticoid and noradrenergic system functioning may play a role. Bertholomey et al. [31] studied the impact of chronic exposure during adolescence to elevated levels of the glucocorticoid stress hormone corticosterone (CORT) on amygdalar function and on the risk of developing AUDS. Adolescent CORT exposure increased alcohol, but not sucrose self-administration, and enhanced stress-induced reinstatement with yohimbine in adulthood. LFQ phosphoproteomic analyses revealed that adolescent CORT exposure resulted in 16 changes in protein phosphorylation in the amygdala, which provided a list of potential novel mechanisms involved in increasing the risk of alcohol drinking. Of particular interest, Bertholomey et al. [31] found that adolescent CORT exposure resulted in increased phosphorylation of the α_2A_ adrenergic receptor (α_2A_AR) mediated by G protein-coupled receptor kinase 2 (GRK2). Bertholomey et al. [31] also found that intra-amygdala infusion of a peptidergic GRK2 inhibitor reduced alcohol seeking, suggesting that GRK2 may provide a novel target for treating stress-induced AUDS. 

As described by Miller et al [32], high-affinity nicotinic acetylcholine receptors containing α4 and β2 subunits (α4/β2* nAChRs, where * denotes other, potentially unidentified subunits) are essential for the rewarding and reinforcing properties of nicotine. α4/β2* nAChRs are ion channel-containing proteins that flux positive ions, including calcium, in response to nicotine or the endogenous neurotransmitter acetylcholine. Activation of α4/β2* nAChRs in the mammalian brain results in the depolarization of neurons on which they are expressed, leading to changes in intracellular signaling, such as the activation of calcium-dependent kinases. Interactions have previously been identified between α4/β2* nAChRs and calcium/calmodulin-dependent protein kinase II (CaMKII) in mouse and human brains [33,34]. Following co-expression of α4/β2 nAChR subunits with CaMKII in human embryonic kidney (HEK) cells, MS/proteomic analyses described by Miller et al. [32] identified eight phosphorylation sites in the α4 subunit. One of these sites and an additional site were identified when α4/β2* nAChRs were dephosphorylated and then incubated with CaMKII in vitro, while three phosphorylation sites were identified following incubation with protein kinase A (PKA) in vitro. Miller et al. [32] then isolated native α4/β2* nAChRs from mouse brain following acute or chronic exposure to nicotine. Two CaMKII sites identified in HEK cells were phosphorylated, and one PKA site was dephosphorylated following acute nicotine administration in vivo, whereas phosphorylation of the PKA site was increased back to baseline levels following repeated nicotine exposure. Although significant changes in β2 nAChR subunit phosphorylation were not observed under these conditions, two novel sites were identified on this subunit, one in HEK cells and one in vitro. 

As described in the Watkins et al. [35] article, reversible protein phosphorylation that modulates neuronal signaling, communication, and synaptic plasticity is controlled by competing kinase and phosphatase activities. Glutamatergic projections from the cortex and dopaminergic projections from the substantia nigra or ventral tegmental area synapse on dendritic spines of specific gamma-aminobutyric acid (GABA)ergic medium spiny neurons (MSNs) in the striatum. Direct pathway MSNs (dMSNs) are positively coupled to PKA signaling and the activation of these neurons enhance specific motor programs, whereas indirect pathway MSNs (iMSNs) are negatively coupled to PKA and inhibit competing motor programs. Psychostimulant drugs increase dopamine signaling and cause an imbalance in the activities of these two programs. While changes in specific kinases, such as PKA, regulate different effects in the two MSN populations, alterations in the specific activity of serine/threonine phosphatases, such as protein phosphatase 1 (PP1), are less well understood. This lack of knowledge partly results from unknown, cell-specific changes in PP1 targeting proteins. Spinophilin is the major PP1-targeting protein in striatal postsynaptic densities. Using MS/proteomics and immunoblotting together with a transgenic mouse expressing hemagglutinin (HA)-tagged spinophilin in dMSNs or iMSNs, Watkins et al. [35] identified novel spinophilin interactions modulated by amphetamine in the different striatal cell types. These results increase our understanding of cell type-specific, phosphatase-dependent signaling pathways that are altered by the use of psychostimulants.

As described by Luxmi et al. [36], identification of enkephalins as endogenous ligands for opioid receptors led to the identification of hundreds of additional bioactive peptides in the nervous systems of species as diverse as *Drosophila* and *Hydra*. The precursors to these neuropeptides have N-terminal signal sequences with multiple potential paired basic amino acid endoproteolytic cleavage sites. Genomic and transcriptomic data from a diverse array of organisms indicated that neuropeptide precursors were present in species lacking neurons or endocrine cells. The enzymes involved in converting neuropeptide precursors into bioactive peptides are highly conserved. The identification of catalytically active peptidylglycine α-amidating monooxygenase (PAM) in *Chlamydomonas reinhardtii*, a unicellular green alga, suggested the presence of a PAM-like gene and peptidergic signaling in the last eukaryotic common ancestor (LECA). Luxmi et al. [36] identified prototypical neuropeptide precursors and essential peptide processing enzymes in the *C. reinhardtii* genome. Positing that sexual reproduction by *C. reinhardtii* requires communication between cells, they used MS to identify proteins in the soluble secretome of mating gametes, and searched for evidence that the putative peptidergic processing enzymes were functional. After fractionation by SDS-PAGE, they identified intact signal peptide-containing proteins as well as those that had been cleaved. The *C. reinhardtii* mating secretome contained multiple matrix metalloproteinases, cysteine endopeptidases, and serine carboxypeptidases, along with one subtilisin-like proteinase. Transcriptomic studies suggest these proteases are involved in sexual reproduction. Multiple extracellular matrix proteins (ECM) were identified in the secretome. Several pherophorins and ECM glycoproteins were present, with most containing typical peptide processing sites, and many had been cleaved, generating stable N- or C-terminal fragments. The Luxmi et al. [36] study suggests that subtilisin endoproteases and matrix metalloproteinases similar to those involved in vertebrate peptidergic and growth factor signaling play an important role in stage transitions during the life cycle of *C. reinhardtii*. Moreover, this study [36] further suggests that endoproteolytic activation of proneuropeptides and growth factors originated in unicellular organisms. The complex endomembrane system in LECA presumably gave rise to the evolution of the preproneuropeptides and growth factors essential for nervous system development and function well before the appearance of neurons.

Despite its low prevalence in the U.S. of ~0.25% [37], schizophrenia (SZ) results in significant health, social, and economic concerns and is one of the 15 leading causes of disability worldwide [38]. Individuals with SZ have an increased risk of premature death with the estimated potential life lost for SZ patients in the U.S. being 28.5 years [39]. As described in the Sowers et al. [40] article, male mice lacking fibroblast growth factor 14 (FGF14) (i.e., *Fgf14*^−/−^) recapitulate key features of SZ, including loss of parvalbumin-positive GABAergic interneurons in the hippocampus, disrupted gamma frequency, and reduced working memory. FGF14 is one of the intracellular FGF proteins that are involved in neuronal ion channel regulation and synaptic transmission. As the molecular basis of SZ and its sex-specific onset are not well understood, the *Fgf14*^−/−^ model may provide a valuable tool to interrogate pathways related to SZ disease mechanisms. Sowers et al. [40] performed LFQ MS to identify enriched pathways in both male and female hippocampi from *Fgf14*^+/+^ and *Fgf14*^−/−^ mice. They found that all of the differentially expressed proteins in *Fgf14*^−/−^ animals, relative to their same-sex wild type counterparts, are associated with SZ, based on genome-wide association data. In addition, differentially expressed proteins were predominantly sex-specific, with male *Fgf14*^−/−^ mice having increased expression of proteins in pathways associated with neuropsychiatric disorders. The Sowers et al. [40] article increases our understanding of the role of FGF14, confirms that the *Fgf14*^−/−^ mouse provides a valuable and experimentally accessible model for studying the molecular basis and gender-specificity of SZ, and also highlights the importance of sex-specific biomedical research.

The articles in the Neuroproteomics Special Issue provide an overview of the unique challenges that must be addressed to carry out meaningful MS/proteomics analyses on neural tissues and the tools and technologies that are available to meet these challenges. The several articles that cover Alzheimer’s disease, addiction, and schizophrenia illustrate how MS/proteomics technologies can be used to help improve our ability to diagnose and understand the molecular basis for neurological diseases. We believe that several of the articles in this Special Issue will be of interest to investigators beyond the field of neurological disorders. In particular, the review by Carlyle et al. [21], “Proteomic Approaches for the Discovery of Biofluid Biomarkers of Neurodegenerative Dementias”, may be of interest to investigators searching for blood and cerebrospinal fluid (CSF) biomarkers for virtually any disease. Similarly, the review by Natividad et al. [25], “From Synapse to Function, A Perspective on the Role of Neuroproteomics in Elucidating Mechanisms of Drug Addiction”, provides a general overview of the utility of MS/proteomics approaches for addressing critical questions in addiction neuroscience that should be equally applicable to investigators involved in virtually any area of biomedical research. Likewise, the article by Wilson et al. [17], “Development of Targeted Mass Spectrometry-Based Approaches for Quantitation of Proteins Enriched in the Postsynaptic Density”, may be useful for any investigator who wishes to design and validate DIA and/or PRM assays for virtually any proteins. Finally, the peroxidase-mediated proximity labeling technology described in the article by Cijsouw et al. [11], “Mapping the Proteome of the Synaptic Cleft through Proximity Labeling Reveals New Cleft Proteins”, may be of interest to investigators interested in mapping many other spatially restricted proteomes. 
